# Blood-Based Biomarkers for the Optimization of Anti-Angiogenic Therapies

**DOI:** 10.3390/cancers2021027

**Published:** 2010-05-28

**Authors:** Cristina Rabascio, Francesco Bertolini

**Affiliations:** Laboratory of Hematology-Oncology, European Institute of Oncology, via Ripamonti 435, Milan 20141, Italy; E-Mail: francesco.bertolini@ieo.it

**Keywords:** cancer, angiogenesis, biomarkers

## Abstract

The dependence of tumor growth and metastasis on blood vessels makes tumor angiogenesis a rational target for therapy. Strategies have been pursued to inhibit neovascularization and to destroy existing tumor vessels, or both. These include direct targeting of endothelial cells, and indirect targeting by inhibiting the release of proangiogenic growth factors by cancer or stromal cells. Many patients benefit from antiangiogenic therapies; thus, development of noninvasive biomarkers of disease response and relapse is a crucial objective to aid in their management. A number of non-invasive tools are described with their potential benefits and limitations. We review currently available candidate biomarkers of anti-angiogenic agent effect. Including these markers into clinical trials may provide insight into appropriate dosing for desired biological effects, appropriate timing of additional therapy, and prediction of individual response. This has important consequences for the clinical use of angiogenesis inhibitors and for drug discovery, not only for optimizing the treatment of cancer, but possibly also for developing therapeutic approaches for various other diseases.

## 1. Introduction

Physiological angiogenesis is distinct from arteriogenesis and lymphangiogenesis and occurs in reproduction, development and wound repair. It is usually focal and self-limited in time. In contrast pathological angiogenesis can persist for years. Pathological angiogenesis is necessary for tumors and their metastases to grow beyond a microscopic size and it can give rise to bleeding, vascular leakage and tissue destruction. These consequences of pathological angiogenesis can be responsible, directly or indirectly, for the symptoms, incapacitation or death associated with a broad range of angiogenesis-dependent diseases such as cancer, autoimmune diseases, age-related macular degeneration and atherosclerosis.

There are important clinical advantages to viewing angiogenesis as an organizing principle: If a clinician recognizes that a patient’s disease might be partly angiogenesis-dependent, it is conceivable that an angiogenesis inhibitor for one type of tumor could be used for a different type of tumor, or even used off-label for a different disease.

Antiangiogenic therapy represents an exciting advance in the clinic management. It primarily targets the activated microvascular endothelial cells in a tumor bed rather than the tumor itself. Anti-angiogenic therapy can also inhibit endothelial cell proliferation and motility indirectly by suppressing a tumor’s production of angiogenic proteins or by neutralizing one of these proteins [1]. Angiogenesis inhibitors are now being approved and introduced into medical practice throughout the world [1]. At the same time, the unmet need for molecular biomarkers has generated an expanding worldwide research effort to develop gene-based and protein-based molecular signatures in blood, platelets and urine, for very early diagnosis of recurrent cancer. There are many clinical uses for biomarkers of angiogenesis, but in addition to developing biomarkers for prediction of response, it will also be important to develop surrogate markers of clinical efficacy because the therapeutic effects can be seen even in the absence of classically defined tumor response [1].

To develop useful biomarkers, one must consider the characteristics of a good biomarker: these include cost-effectiveness, low baseline levels in normal individuals, accessibility by noninvasive means such as blood robustness in the clinical setting, and reproducibility in multiple clinical centers. Currently, there are several candidates for noninvasive biomarkers of antiangiogenic therapy that can be assessed in patient blood.

## 2. Angiogenic Growth Factors, Soluble Receptors and Other Proteins

Vascularized tumor masses usually express multiple angiogenic factors and cytokines. Tumor hypoxia modulates not only angiogenic factor expression levels, but also the ratio of, and interplay between, various angiogenic factors. These tumor-derived angiogenic factors often synergistically stimulate angiogenesis. For example, crosstalk between VEGF and several other signaling systems, including members of the platelet-derived growth factor (PDGF), fibroblast growth platelet-derived growth factor (FGF), angiopoietin (Ang), and SDF 1, have been shown to lead to alterations in angiogenesis and vasculogenesis as well as vascular remodeling, maturation, and stability [2,3,4,5,6,7,8]. Non-VEGF angiogenic factors such as PDGF-BB and FGF-2 have been suggested to reciprocally interact in vascular cells to modulate angiogenesis, vascular remodeling, maturation, and stability [9]. Notably, PDGF receptors are usually not detectable in quiescent endothelial cells. However, after exposure to FGF-2, expression levels of PDGFRs are highly elevated via transcriptional regulation, and endothelial cells gain a robust response to PDGF ligands [9]. This suggests that angiogenic factors can aberrantly increase endothelial cell response to other factors. The role of PDGF-BB in tumor angiogenesis remains controversial. Most studies have shown that blockade of PDGF signaling is crucial for antiangiogenic cancer therapy [10,11], but a recent study proposed the opposite effect of PDGF blockade on tumor angiogenesis [12]. These investigators suggested that because PDGF-BB promotes maturation and prevents leakiness of the primitive vasculature, it may therefore be critical for drug delivery. This controversial hypothesis warrants further investigation. Angiogenic growth factors and soluble VEGF receptors such as VEGFR1, VEGFR2 and VEGFR3 are currently being investigated in a variety of plasma samples using ELISA methodology. Associations between outcomes of antiangiogenic therapy with VEGF levels in the circulation has been reported in some phase II studies [13,14,15] but many studies have shown a lack of correlation between VEGF levels at baseline and outcome of antiangiogenic therapy [16,17]. Circulating levels of soluble VEGFR2 and VEGFR3 proteins are decreased by tyrosine kinase inhibitors (TKIs) [18,19,20,21,22,23] that directly target these VEGF receptors but not by bevacizumab [24,25]. The mechanisms by which these changes occur, their biological significance value, are not understood. For example, it was shown in renal cancer patients receiving the tyrosine kinase inhibitor sunitinib, circulating levels of VEGF-A in the blood increased during each cycle of treatment, whereas soluble VEGFR2 decreased within two weeks after treatment stopped, the levels of these biomarkers returned to near basal levels but the respective changes could be induced again during the next (4 week) cycle of daily therapy [22]. In some clinical trials baseline levels of VEGF-A and or soluble VEGFR3 and VEGF-C have shown a predictive potential in sarcoma, lung, and kidney cancer patients receiving anti angiogenic drugs, alone or in combination with chemotherapy [19,20,26,27,28]. While the use of circulating VEGF as a biomarker remains unclear, evaluation of the VEGF genotype has emerged as a predictive biomarker candidate from the phase III study of bevacizumab with chemotherapy versus chemotherapy alone in patients with metastatic breast cancer. Some particular VEGF genotypes, namely VEGF-2578AA and VEGF-1154A, were associated in advanced breast cancer patients to a superior median OS [29]. Exploration of biomarkers other than VEGF members is critical given their known involvement in tumor angiogenesis and vessel maturation. Soluble ICAM1 was an independent prognostic factor in one study evaluating lung cancer patients treated with bevacizumab and chemotherapy [26]. In previously untreated patients with metastatic colorectal cancer responses to vatalanib plus chemotherapy correlated directly with tissue messenger RNA levels of VEGFR1, LDHA (lactate dehydrogenase A) and Glut1 and inversely with hypoxia-inducible factor 1α (HIF1α1) [30]. Certain inflammatory cytokines might have potent proangiogenic effects: the IL-8A-215T polymorphism (associated with an increase in IL-8 expression) was found to be a possible predictor of response to the association of bevacizumab and chemotherapy in ovarian cancer [31]. In colorectal cancer patients treated with chemotherapy plus bevacizumab, high levels of baseline IL-8 were predictive of a shorter PFS [32]. An interesting rather new tissue biomarker is phospho-VEGFR2: in patients with inflammatory breast cancer, anti-angiogenic therapies resulted in a reduction of phospho-VEGFR2, associated with an increase in tumor cell apoptosis and no change in tumor cell proliferation [33]. These results emphasize the necessity of evaluating the predictive biomarkers in a dynamic manner, that is, before and soon after the beginning of antiangiogenic treatment. Sometimes the use of cytokines and angiogenic growth factors as measures of angiogenic activity can be complicated by the fact that platelets contain and could release many angiogenic and antiangiogenic factors that could confound accurate measurements in patient samples. More work is needed to ascertain whether these biomarkers can predict patients’ survival or response to antiangiogenic therapies [1,34,35].

## 3. Molecular Markers

The transcriptome of endothelial cells purified from cancer patients has been investigated by different laboratories. Genetic signatures should still be fully validated in the clinical setting, but they are potentially important also for the development of therapeutics specifically targeting tumor vessels [36].

Only a small number of genes is considered to be endothelial-restricted or endothelial-specific. One of these is VE-cadherin. It is commonly accepted that contact inhibition of cell proliferation is at least partially mediated by the establishment of cadherin-based junctions. Endothelial cell division is inhibited when cells are plated onto a substrate containing the VE-cadherin extracellular domain [37], indicating that VE-cadherin engagement limits endothelial cell proliferation. VE-cadherin associates with VEGFR2 upon VEGF induction or angiogenic stimulation [38,39] VEGF transduces a survival signal to endothelial cells through a VE-cadherin-dependent mechanism. This signal needs VEGF R2-VE-cadherin association [38]. More recently, tumor angiogenesis could be blocked by antibodies against VE-cadherin, suggesting that VE-cadherin activity is necessary for vascular proliferation in adults [40,41]. VE-cadherin was initially considered as a constitutive protein with unregulated expression. However, this issue has to be reconsidered in view of several recent studies: Endothelial cells from human breast carcinoma contained elevated amounts of VE-cadherin mRNA compared to normal mammary vasculature [42] and the number of copies of VE-cadherin transcripts in the blood of different cancer patients is significantly increased when compared to healthy controls. Quantitative RT-PCR copies for VE-cadherin have been shown to correlate with the number of circulating endothelial cells and their viability status [43]. This evaluation offers some distinct advantages: VE-cadherin RNA enumeration can be performed in large series of frozen samples and inter-laboratories standardization seems to be more easily achievable. However, very few data about measurement of RNA copies in patient peripheral blood samples are as yet available. Moreover, the optimal collection tube and processing method for RNA purification is still being optimized and the methodology has to be standardized.

## 4. Cellular Markers

A promising area of antiangiogenic monitoring is the measurement of circulating endothelial cells (CECs) and endothelial precursor cells (CEPs) in the peripheral blood of patients.

CEPs (primarily derived from bone marrow) significantly contribute to tumor angiogenesis [44,45] and antivasculogenic agents inhibit mobilization or integration of circulating endothelial cells into tumor angiogenic vessels to impair tumor growth [45,46]. In healthy subjects, CECs are a very rare cell population representing 1/1,000–100,000 of circulating blood cells [35]. In many pathological conditions the number of CECs is increased [47]. CEC and CEP levels are increased in the peripheral blood of patients affected by some types of cancer, and return to normal values in patients undergoing complete remission [35].

CEC/CEP numbers and viability have been measured in different clinical trials involving cancer patients treated with various anti-angiogenic therapies [48,49,50,51,52].

The majority of CECs shows characteristics of mature, terminally differentiated and frequently apoptotic cells, only a subpopulation of which expresses antigens that suggest a stem or progenitor-like phenotype. These putative circulating endothelial progenitors (CEPs) might be home to sites of active vasculogenesis.

Differentiating CEPs from CECs based on different expression of surface molecules is very difficult due to the antigenic promiscuity among hematopoietic cells and progenitors, platelets, CECs and CEPs. In fact, there is no single antigen able to discriminate between CECs, platelets and hematopoietic cells [35]. Multiparametric flow-cytometry is thus used for CEC and CEP enumeration. Endothelial cells are identified by the expression of markers such as CD31, CD146 or VEGFR2; CD45 expression is used to exclude hematopoietic cells from the analysis. The use of a nuclear staining for DNA is crucial to exclude aggregated platelets and/or endothelial micro and macro particles from the CEC count [34,35,53,54,55,56,57,58]. How to discriminate CEPs in the CEC population is still a matter of controversy. Methodological inconsistency between flow cytometry procedures, involving differences in the combinations of markers, gating strategies, and the occasional use of a pre‑enrichment step, has led to different CEC values reported in the literature [35]. Thus, there is a need for standardization of flow cytometry procedures to minimize intra- and inter-laboratory variability [58].

## 5. CECs as Surrogate Markers of Angiogenesis and Anti-angiogenic Drug Activity in Medical Oncology

CEC levels are increased in the peripheral blood of patients affected by some types of cancer [49,50,51,59]. In metastatic breast cancer, patients treated with low dose metronomic chemotherapy using CTX and methotrexate, the CEC count after two months of continuous (daily) therapy, was a particularly good predictor of disease-free and overall survival after a follow-up of more than two years. Patients showing a CEC count above physiological levels after two months of therapy had a significantly improved progression-free and overall survival [48].

When the humanized anti-VEGF antibody bevacizumab was added to the metronomic chemotherapy for the treatment of metastatic breast cancer, patients who showed a clinical response in a phase II clinical trial (as well as a larger population of patients who had a clinical benefit from the treatment) had significantly greater baseline levels of viable CECs than did patients who failed to respond; furthermore, the number of apoptotic CECs before therapy initiated was associated with prolonged progression-free survival [51]. 

In patients treated with the small molecule anti-angiogenic agent sunitinib, changes in CECs differed between the patients with clinical benefit and those with progressive disease [34]. In a study where locally advanced patients received regular-dose chemotherapy, plus endocrine therapy plus bevacizumab before surgery, baseline CEP count was positively associated with a clinical response [52]. 

Taken together, our studies indicate that assessment of CECs might be an estimation tool for prediction of response in patients with advanced breast cancer receiving metronomic chemotherapy alone or in association with bevacizumab. The increased number of CECs in patients receiving a clinical benefit from metronomic chemotherapy was due to an increased number of apoptotic CECs. This finding demonstrates the anti-vascular activity of metronomic chemotherapy. The measurement of CECs, of their viability, and of CEC subpopulations (e.g., VEGFR2 + CECs, activated CD105 + CECs, or VEGFR3 + CECs, possibly involved in lymphangiogenesis) might be useful for futures studies on news antiangiogenic drugs, alone or in combination with chemotherapeutics. When considering that in undifferentiated patient pools, the number of non-responders could impair a trial's endpoint, CEC-related measurements might also help in identifying responders and non-responders to a given therapeutic regimen including anti-angiogenic drugs. This patient stratification may significantly reduce the costs of novel antiangiogenic cancer therapies by targeting treatments to those patients most likely to benefit.

Conflicting studies showing CEP contribution to tumor vessels [60,61] have been recently explained by the findings that CEP contribution to the tumor vasculature depends on variables such as tumor stage and grading [62,63].

CEPs might be relevant in tumor relapse after chemotherapy or anti-vascular treatment [49,64,65,66] and this might explain how antiangiogenic drugs can increase chemotherapy efficacy [66]. In addition to CEPs, other marrow-derived circulating cell populations might be involved in tumor angiogenesis but these cells might be investigated as biomarkers, if enumeration procedures are clinically validated [19].These possibilities await confirmation in prospective randomized clinical trials.

## 6. Blood Pressure as a Biomarker

Another predictive indirect blood and pharmacodynamic biomarker factor, emerging during the therapy, is the increase in blood pressure (hypertension) observed in some cancer patients receiving anti-VEGF therapies. Several studies [20,67,68,69,70] have found that the degree of hypertension might correlate with survival after therapies including bevacizumab or the small molecule axitinib. The Eastern Cooperative Oncology Group study 4599, for instance, evaluated patients with lung cancer treated with a combination of chemotherapy plus bevacizumab. Hypertension was defined as blood pressure >150/100 at any previous time or at least a 20-mmHg increase in diastolic blood pressure from baseline. In a multivariate analysis, hypertension was associated with a favorable clinical outcome [38]. In breast cancer patients receiving taxanes and bevacizumab the VEGF-634CC and VEGF-1489TT genotypes were found to be associated with reduced risk of grade 3–4 hypertension [70].

## 7. Conclusions and Future Perspectives

Surrogate biomarkers of angiogenesis are urgently needed to better design preclinical studies and clinical trials involving antiangiogenic drugs, alone or in association with other therapies. With increasing numbers of antiangiogenic agents being approved, or considered for approval, the need for biomarkers is more critical than ever for efficacy, safety, and cost considerations. Preliminary biomarker data are emerging: VE-cadherin RNA, flow cytometric CEC and CEP enumeration will offer non-overlapping clinical information, and a multifaceted evaluation of these surrogate markers will be tested in large, well-designed, prospective clinical trials. Biomarker selection would be greatly supported if we achieved a better understanding of the mechanism of action of these agents in cancer patients. Finally, once the candidate biomarkers are identified, standardized techniques will be required to measure them. Although many challenges remain, future validation of biomarkers and their incorporation into clinical practice holds promise for improved cancer treatment with antiangiogenic agents. Now, a collaborative effort among clinicians, pharmaceutical companies, governmental agencies and private foundations is needed to realize this goal.
